# Discrimination of circular polarized light

**DOI:** 10.1038/s41377-022-00839-z

**Published:** 2022-05-30

**Authors:** Guangbo Qu, Guibin Jiang

**Affiliations:** 1 State Key Laboratory of Environmental Chemistry and Ecotoxicology, Research Center for Eco-Environmental Sciences, Chinese Academy of Sciences, 100085 Beijing, China; 2 School of Environment, Hangzhou Institute for Advanced Study, University of Chinese Academy of Sciences, 310024 Hangzhou, China; 3 Institute of Environment and Health, Jianghan University, 430056 Wuhan, China; 4 University of Chinese Academy of Sciences, 100049 Beijing, China

**Keywords:** Optical materials and structures, Applied optics

Nature Nanotechnology **17**, 408–416 (2022)

10.1038/s41565-022-01079-3

The polarization control of light opens numerous explorations like information communication or three-dimensional displays. However, because the difference between number of left-handed and right-handed photons in the free electromagnetic field is less than a percent, there is still a great challenge to measure the difference.

Researchers from Jiangnan University, together with their international collaborators present a versatile method to recognize the polarization state of light by using chiral gold nanoparticle film deposited onto anodized aluminum oxide substrates. In integrated photonic devices, the photocurrent is directly related to the polarization state of light with 2.41-fold difference between right-handed circularly polarized and left-handed circularly polarized light, which provides advantages over their inorganic or organic chiral film counterparts by 1–2 orders of magnitude. The concept is clarified referring to the wave-guided chiroplasmonic modes propagating along the film surface and charge entrapment in phenylalanine on gold nanoparticles.

